# Taxpayers' perceptions and behavioral responses to GST 2.0 slab rationalization: evidence from MSMEs in the Indian automobile industry

**DOI:** 10.3389/fpsyg.2026.1885139

**Published:** 2026-07-21

**Authors:** R. Muthu Sundari, N. Saravanabhavan

**Affiliations:** Department of Commerce, Vellore Institute of Technology, Vellore, Tamil Nadu, India

**Keywords:** GST 2.0, MSME stakeholders, PLS-SEM, slippery slope framework, tax morale, theory of planned behavior, voluntary tax compliance behavior

## Abstract

**Introduction:**

Tax reforms in emerging economies are increasingly being conceptualized as a tool of behavioral governance as opposed to being a tool of fiscal restructuring. This empirical research undertakes the behavioral implications of the GST 2.0 slab rationalization on voluntary tax compliance among Micro, Small and Medium Enterprises (MSMEs) that are engaged in the automotive market of India. Drawing on an integrated theoretical construct incorporating the Theory of Planned Behavior (TPB Theory), Tax Morale Theory (TM Theory), and the Slippery Slope Framework (SSF), the current research incorporates a mechanism-based model that outlines the extent to which cognitive appraisals, intrinsic moral motivation, and institutional trust all have an impact on compliance behavior.

**Methods:**

The survey data collected among the key stakeholders of MSMEs located in the main automotive industrial belts of Tamil Nadu, and analyzed through Partial Least Squares Structural Equation Modeling (PLS-SEM) were used as empirical evidence.

**Results:**

Findings suggest that positive attitudes and perceived social norms are not sufficient to reflect into voluntary compliance but instead, perceived behavioral control is the most salient antecedent, and Tax Morale is a pivotal mediating construct. Furthermore, Trust in tax authorities strengthens the relationship between subjective norms and tax morale, highlighting the importance of institutional credibility in shaping voluntary compliance behavior. Overall, the findings suggest that perceived capability, intrinsic moral motivation, and institutional trust jointly influence compliance behavior under GST 2.0.

**Discussion:**

The study contributes to behavioral taxation literature by integrating cognitive, moral, and institutional perspectives within a unified framework and offers practical implications for designing trust-based tax reforms in emerging economies.

## 1 Introduction

Tax reform is increasingly viewed as a behavioral intervention in contemporary fiscal policy rather than merely an administrative or revenue-generating tool. Modern fiscal theory emphasizes that the effectiveness of tax reforms is influenced not only by their design and implementation but also by taxpayers' perceptions, understanding, and behavioral responses to institutional change (Feld and Frey, 2007; Kirchler et al., 2008; Alm, 2019). In that regard, voluntary tax compliance has become the primary issue of scholarly and policy concern, as it has become the pillar of sustainable government revenue and an additional contributor to the enforcement of the fiscal social contract (Alm and Torgler, 2011; OECD, 2020; Appiah et al., 2024). The behavioral public finance also highlights the fact that the perceived fairness, trust, and institutional legitimacy influence compliance decisions, thus not just limiting to the explanations of deterrence (Luttmer and Singhal, 2014; Torgler, 2007; Dimitras et al., 2025).

In this paradigm of behavior, rationalization and simplification of tax rates are broadly discussed as the main reform tools which are aimed to decrease compliance costs of taxpayers, improve transparency and increase taxpayer confidence (Evans, 2003; Saad, 2014). However, the empirical experience shows that the perception of fairness can be undermined when complex tax systems are present (i.e., with several rate slabs, frequent changes, and vague categorizations), which reduces the Tax Morale (TM Theory) and discourages self-compliance, especially in the case of small enterprises with limited administrative resources (Loo et al., 2010; Alm, 2019; Tumoro and Pandya, 2025). These results highlight the need to question behavioral reactions of reforming taxation in particular institutional and sectoral settings.

One of the biggest indirect tax reforms to have been made in a developing economy is the introduction of the Goods and Services Tax (GST) in India in 2017. This reform was also to streamline the taxation framework and do away with the cascading effects and consolidate various types of central and state indirect taxes into a single framework (Mukherjee, 2020). The later experience, however, showed that there are still consistent challenges, such as a multi-slab rate system, frequent change of rates, classification controversy, inverted duty frameworks, and high compliance expenses (Kapoor and Singh, 2025; Government of India, 2022). These complexities have also led to compliance fatigue, increased uncertainty, and reduced trust in taxpayers and therefore there are concerns on whether voluntary compliance can be sustained over the long run.

In response, policymakers have proposed GST 2.0, in which slab rationalization is one of the major goals. The proposed GST 2.0 framework aims to harmonize GST rates with the 5% and 18% rates, reduce ambiguity, reverse the structure of duties, and simplify compliance procedures (Government of India, 2022; Reserve Bank of India, 2025; Thakur and Devi, 2025). As a proposed institutional reform, GST 2.0 is expected to influence perceptions of fairness, legitimacy, and institutional credibility, which are key determinants of voluntary tax compliance (VTC) (Tyler, 2006; Feld and Frey, 2007; OECD, 2020).

Micro, Small and Medium Enterprises (MSMEs) constitute an important pillar of the Indian economy and are particularly vulnerable to indirect tax reforms. Empirical data show that MSMEs pay disproportionately high compliance costs due to the lack of tax knowledge, technological shortages, and limited administrative ability (OECD, 2020; Dimitras et al., 2025). The automobile industry is strategically important due to its complex supply chain and extensive GST exposure, making automobile MSMEs particularly sensitive to the anticipated effects of GST 2.0 slab rationalization.

Existing studies have largely examined GST from fiscal and economic perspectives. Prior research suggests that GST has improved tax administration efficiency, enhanced revenue mobilization, broadened the indirect tax base, and promoted greater formalization of economic activities in India (Mukherjee, 2020; Garg et al., 2024a,b,c,d). Several studies also report that GST has contributed to improved interstate trade integration, reduced cascading tax effects, and strengthened market efficiency by creating a unified national tax framework (Garg et al., 2024a,b,c,d; Mukherjee, 2020). Moreover, comparative evidence indicates that GST has enhanced indirect tax revenue mobilization efficiency across Indian states relative to the previous VAT regime (Mallick, 2026). Recent studies suggest that GST revenue performance and efficiency are influenced by macroeconomic conditions, policy design, and administrative effectiveness (Garg et al., 2024a,b,c,d). While these studies provide valuable evidence regarding the fiscal and macroeconomic outcomes of GST implementation, they predominantly focus on aggregate economic indicators and administrative performance. However, these studies provide limited insight into how taxpayers perceive GST reforms and how such perceptions influence voluntary tax compliance behavior, particularly in the context of GST 2.0 slab rationalization among MSMEs.

Despite the growing body of GST compliance research in India, existing studies have primarily focused on GST awareness, GST knowledge, compliance costs, GST adoption, taxpayer compliance behavior, and GST revenue efficiency (Garg et al., 2024a,b,c,d; Mukherjee, 2020; Kapoor and Singh, 2025). Kapoor and Singh (2025) reported that GST compliance costs and procedural complexities remained important challenges for small businesses despite improvements in tax administration. Sharma et al. (2023) found that GST knowledge and technological readiness significantly influence compliance outcomes among Indian taxpayers. Similarly, Garg et al. (2024a,b,c,d) demonstrated that administrative simplicity and taxpayer awareness positively affect GST compliance behavior. Collectively, these studies indicate that operational and compliance-related factors play an important role in GST compliance decisions. However, limited attention has been devoted to understanding how taxpayers' cognitive evaluations, tax morale, and trust in tax authorities jointly influence voluntary tax compliance behavior under GST 2.0 slab rationalization. Although the Theory of Planned Behavior (Ajzen, 1991), Tax Morale Theory (Torgler, 2007; Alm and Torgler, 2011), and the Slippery Slope Framework (Kirchler et al., 2008; Gangl et al., 2015) have individually received substantial empirical support, their integrated application in the context of GST 2.0 slab rationalization remains largely unexplored.

Addressing these gaps, the present study develops and empirically tests an integrated behavioral framework that combines TPB, Tax Morale Theory, and the Slippery Slope Framework to explain voluntary tax compliance behavior among MSMEs under GST 2.0 slab rationalization. The study contributes theoretically by explaining how cognitive evaluations are translated into voluntary tax compliance through tax morale under varying levels of institutional trust, while providing empirical evidence from automobile-sector MSMEs operating under the GST 2.0 reform context.

Based on this, the research questions to be explored under the study are:

**RQ1:** How do attitude, subjective norms, and perceived behavioral control influence voluntary tax compliance behavior?
**RQ2:** How do attitude, subjective norms, and perceived behavioral control influence tax morale?
**RQ3:** Does tax morale mediate the relationship between TPB constructs and voluntary tax compliancebehavior?
**RQ4:** Does trust in tax authorities moderate the relationships between attitude, subjective norms, perceived behavioral control, and tax morale?

The remainder of this document is structured as follows. In Section 2, the relevant literature is reviewed, theoretical foundations are identified, and hypotheses are proposed. The research strategy, which includes data collection and PLS-SEM analysis, is presented in Section 3. Section 4 provides an introduction to the study's empirical findings, while Section 5 discusses the findings and their consequences. A conclusion on limits and future research directions concludes Section 6.

## 2 Literature review, theoretical framework, and hypothesis development

Voluntary tax compliance (TC) is fundamental to sustainable public finance because it reduces administrative costs and stabilizes government revenue (Allingham and Sandmo, 1972; Alm, 2019). While early compliance research emphasized deterrence, contemporary studies increasingly recognize the importance of psychological and social determinants of compliance behavior (Kirchler et al., 2008; Torgler, 2007). Tax reforms that alter tax rates, complexity, or perceived fairness can significantly influence taxpayer behavior (Feld and Frey, 2007; Saad, 2014). In India, GST slab rationalization seeks to enhance fairness and compliance; however, empirical evidence regarding MSMEs' behavioral responses remains limited (OECD, 2019; Loo et al., 2010; Kapoor and Singh, 2025).

Moreover, existing GST studies have predominantly examined operational, compliance, and fiscal dimensions of GST implementation, including taxpayer awareness, GST adoption, compliance costs, compliance behavior, and revenue efficiency (Garg et al., 2024a,b,c,d; Mukherjee, 2020; Kapoor and Singh, 2025). Prior GST compliance studies have reported that taxpayer awareness, GST knowledge, compliance costs, technological readiness, and administrative complexity significantly influence compliance behavior among Indian businesses (Kapoor and Singh, 2025; Sharma et al., 2023; Garg et al., 2024a,b,c,d). Despite their contributions, these studies provide limited understanding of the psychological mechanisms through which GST reforms influence voluntary tax compliance.

To address this gap, the study develops an integrated behavioral framework for explaining voluntary tax compliance behavior among MSME taxpayers under GST 2.0 slab rationalization.

### 2.1 Theoretical foundations

This study adopts an integrated theoretical framework comprising the Theory of Planned Behavior (TPB), Tax Morale Theory (TM), and the Slippery Slope Framework (SSF) to explain taxpayers' behavioral responses to GST 2.0 slab rationalization. While TPB provides the primary behavioral foundation, the present study adopts a modified TPB perspective by focusing on the direct effects of attitude, subjective norms, and perceived behavioral control on tax morale and voluntary tax compliance behavior. Furthermore, the study focuses on the trust dimension of the Slippery Slope Framework because voluntary compliance is more closely associated with institutional legitimacy and trust than with enforcement-based compliance mechanisms. Accordingly, TPB explains cognitive evaluations, Tax Morale Theory explains their translation into moral motivation, and SSF highlights the role of institutional trust, together providing a behavioral explanation of voluntary tax compliance under GST 2.0 slab rationalization.

#### 2.1.1 Theory of planned behavior (TPB)

The TPB Theory indicates that attitude, subjective norms, and perceived behavioral control are important determinants of compliance behavior (Ajzen, 1991). The framework has been widely used to examine tax compliance choices (Richardson, 2006; Bobek et al., 2007; Garg et al., 2024a,b,c,d). The attitude constitutes outcome evaluation, subjective norms reflect a perceived social pressure and the perceived behavioral control reflects a perceived ease of compliance (Hanno and Violette, 1996; Loo et al., 2010). The positive role of these constructs in tax compliance behavior is corroborated by empirical evidence (Bobek and Hatfield, 2003; Alm, 2019). Nevertheless, the TPB Theory does not focus on moral and institutional processes that play a vital role in the tax reforms, including GST slab rationalization (Alm and Torgler, 2011; Sniehotta et al., 2014).

#### 2.1.2 Tax morale theory (TM Theory)

TM Theory refers to taxpayers' intrinsic motivation to comply with tax obligations based on ethical values, social norms, and perceptions of fairness and legitimacy (Torgler, 2007; Frey and Torgler, 2007). A substantial amount of literature confirms that TM is a strong predictor of voluntaristic compliance, and it is often more effective than deterrence-based mechanisms (Alm, 2019). Its importance is particularly high in developing economies, where the enforcement possibilities are limited (Dell'Anno, 2009). Empirical researches show that positive attitudes toward the tax system, distributive justice and social norms have a positive impact on TM (Feld and Frey, 2007; Alm and Torgler, 2011). Moral accountability is also increased by the perceptions of justice, openness, and simplicity (Gangl et al., 2015). Under the framework of GST 2.0 slab rationalization, simplified rate systems are expected to work toward the strong perception of fairness and hence the increase in TM amongst MSMEs. The TM is thus theorized as a mediating variable that links taxpayers' cognitive evaluations with voluntary compliance behavior (Dell'Anno, 2009; Alm, 2019).

#### 2.1.3 Slippery slope framework (SSF)

The Slippery Slope Framework posits that tax compliance is influenced by both trust in tax authorities and the perceived power of authorities (Kirchler et al., 2008). Trust also encourages cooperation, voluntary compliance, on the other hand, over-coercion promotes non-internalized and enforced compliance (Muehlbacher et al., 2011; Gangl et al., 2015). Perceptions of fairness, competence and ethical behavior are mirrored by trust which are especially relevant in reforms like rationalization of GST slabs. The high trust levels strengthen the moral obligation and voluntary compliance (Feld and Frey, 2007; Kirchler et al., 2008). In this regard, trust in tax authorities is conceptualized as a moderating factor that strengthens the relationships between TPB constructs and tax morale.

### 2.2 TPB constructs and voluntary compliance behavior

Attitude, subjective norms, and perceived behavioral control are the core TPB constructs influencing voluntary tax compliance behavior. Positive perceptions of fairness, social expectations, and confidence in compliance capabilities encourage taxpayers to comply voluntarily, particularly under simplified GST frameworks (Ajzen, 1991; Bobek et al., 2007; Alm, 2019). Within the framework of rationalization of the Goods and Services Tax (GST) slabs, simplified rates and the corresponding decrease in compliance costs are likely to encourage voluntary compliance behavior, especially among MSMEs that are constrained by administrative forces (OECD, 2020; Garg et al., 2024a,b,c,d).

Attitude toward GST compliance reflects taxpayers' evaluation of the rationalized framework (Hanno and Violette, 1996; Loo et al., 2010). Subjective norms capture perceived social expectations regarding compliance, while perceived behavioral control reflects taxpayers' confidence in fulfilling tax obligations (Bobek et al., 2007; Torgler, 2007). Together, these factors are expected to positively influence voluntary tax compliance behavior (Saad and Ya'u, 2019; Alm, 2019).

**H1a:** Attitude has a positive impact on Voluntary tax compliance behavior.
**H1b:** Subjective norms have a positive impact on Voluntary tax compliance behavior.
**H1c:** Perceived behavioral control has a positive impact on Voluntary tax compliance behavior.

### 2.3 TPB constructs and tax morale

TM is the intrinsic motivation to meet tax obligations and cognitive processes and social factors play a crucial role in the formation of the TM. Based on the TPB Theory, previous studies suggest that the attitude, subjective norms, and perceived behavioral control are considered the key determinants of TM as they create moral judgments, social expectations, and the perceived ability to comply with tax laws (Ajzen, 1991; Torgler, 2007; Kirchler et al., 2008).

Positive attitudes toward taxation are expected to strengthen taxpayers‘ moral obligation to comply because favorable evaluations of the tax system reinforce perceptions of fairness and civic responsibility (Bobek and Hatfield, 2003; Alm and Torgler, 2011). Subjective norms may enhance tax morale by encouraging individuals to internalize socially accepted compliance behavior and align their actions with prevailing social expectations (Bobek et al., 2007; Torgler, 2007). Perceived behavioral control can strengthen moral motivation by increasing taxpayers' confidence in their ability to fulfill tax obligations and comply with tax requirements effectively (Ajzen, 1991; Loo et al., 2010; Garg et al., 2024a,b,c,d). Therefore, TPB constructs are expected to positively influence tax morale.

**H2a:** Attitude has a positive impact on Tax morale.
**H2b:** Subjective norms have a positive impact on Tax morale.
**H2c:** Perceived behavioral control has a positive impact on Tax morale.

### 2.4 Tax morale and voluntary compliance behavior

Tax morale (TM), defined as taxpayers' intrinsic motivation and moral obligation to comply with tax laws, is widely recognized as a key antecedent of voluntary tax compliance behavior (Cummings et al., 2009; Alabede et al., 2011; Mebratu, 2024). Prior studies suggest that taxpayers with higher levels of tax morale are more willing to fulfill their tax obligations voluntarily because they perceive compliance as a moral and civic responsibility rather than merely a legal requirement (Alm and Torgler, 2011; Richardson, 2006). Accordingly, higher levels of tax morale are expected to promote voluntary tax compliance behavior.

**H3:** Tax morale has positive impact on Voluntary tax compliance behavior.

### 2.5 Mediating role of tax morale

Although TPB Theory explains taxpayers' cognitive evaluations, it provides limited explanation regarding how these evaluations are translated into voluntary compliance behavior. TM Theory fills this gap since it describes the inherent moral drive underpinning compliance behavior (Torgler, 2007; Frey and Torgler, 2007). The more taxpayers feel a moral responsibility toward compliance, the more likely they are to engage in voluntary compliance behavior (Alm, 2019). Based on this, TM is conceptualized as an intervening variable between TPB constructs and voluntary tax compliance behavior.

**H4a:** Tax morale mediates the relationship between Attitude and Voluntary tax compliance behavior.
**H4b:** Tax morale mediates the relationship between Subjective norms and Voluntary tax compliance behavior.
**H4c:** Tax morale mediates the relationship between Perceived behavioral control and Voluntary tax compliance behavior.

### 2.6 Moderating value of trust among tax authorities

Trust in tax authorities plays an important role in strengthening taxpayers' moral motivation toward voluntary compliance. The Slippery Slope Framework suggests that taxpayers who place their trust in authorities will more readily convert moral motivation into compliant behavior due to the fact that trust serves to strengthen compliance with norms and the perceived legitimacy (Kirchler et al., 2008; Gangl et al., 2015). Trust in tax authorities is conceptualized as a moderator that strengthens the relationships between TPB constructs and tax morale by enhancing perceptions of fairness, legitimacy, and institutional credibility.

Higher levels of trust increase perceptions of fairness, transparency, and institutional legitimacy, thereby encouraging taxpayers to internalize compliance-related beliefs and convert them into stronger moral commitment toward tax compliance (Feld and Frey, 2007; Gangl et al., 2015).

**H5a:** Trust in tax authorities moderates the relationship between Attitude and Tax morale.
**H5b:** Trust in tax authorities moderates the relationship between Subjective norms and Tax morale.
**H5c:** Trust in tax authorities moderates the relationship between Perceived behavioral control and Tax morale.

### 2.7 Conceptual framework

**Figure 1:**
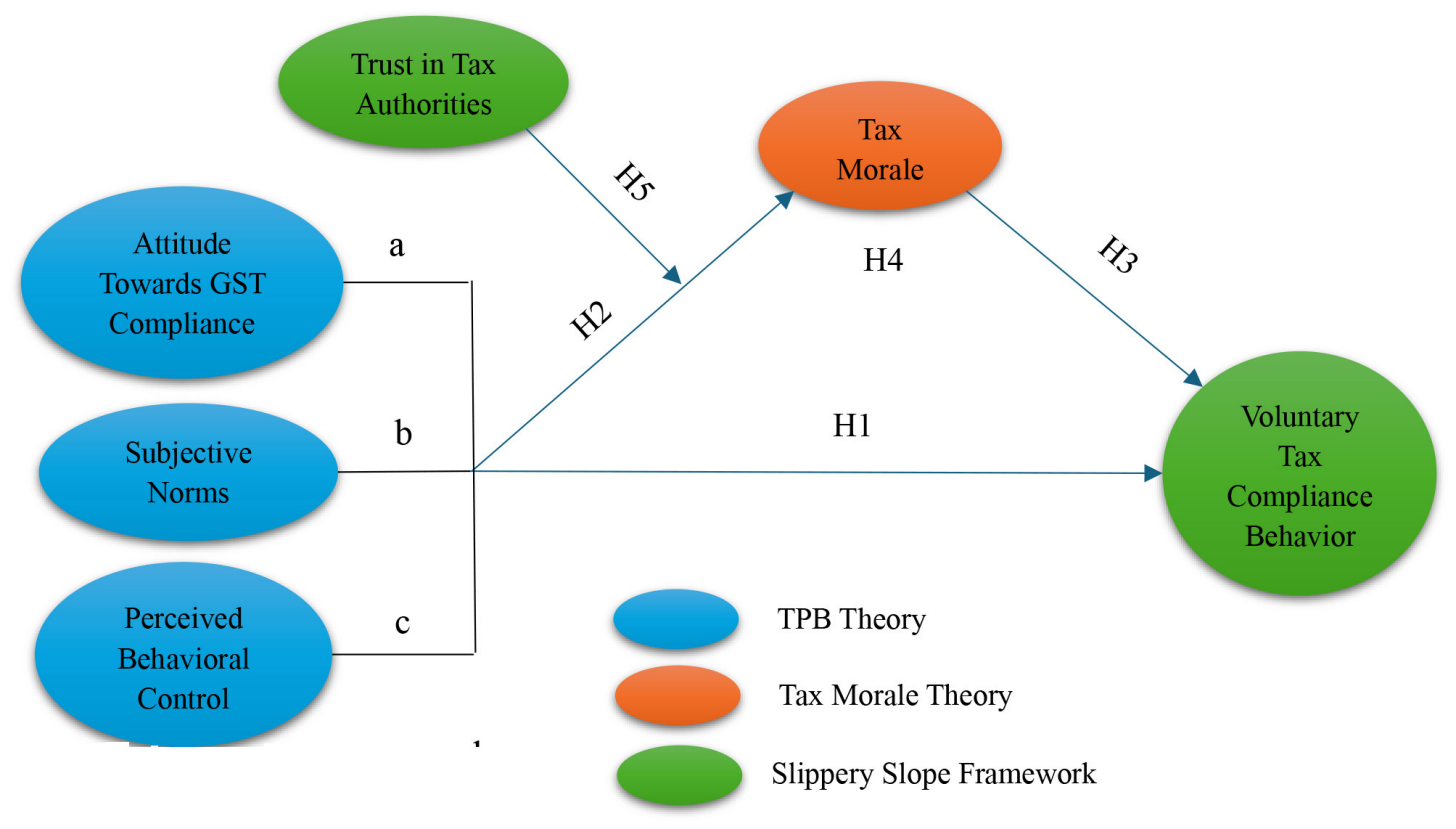
Conceptual framework.

The proposed framework integrates TPB, Tax Morale Theory, and the Slippery Slope Framework to explain voluntary tax compliance under GST 2.0 slab rationalization by combining cognitive, moral, and institutional dimensions of taxpayer behavior. In line with TPB, attitude, subjective norms, and perceived behavioral control are conceptualized as cognitive antecedents of tax morale and voluntary tax compliance behavior (Ajzen, 1991; Bobek et al., 2007). TM Theory explains how cognitive evaluations are translated into voluntary compliance behavior, as a mediating variable that determines intrinsic moral motivation among taxpayers (Torgler, 2007; Alm, 2019). In addition, the moderating role of trust in tax authorities strengthens the relationships between TPB constructs and tax morale by enhancing perceptions of legitimacy and institutional credibility (Kirchler et al., 2008; Gangl et al., 2015). The conceptual model is described in Figure 1.

## 3 Methodology

The proposed research model (Figure 1) was empirically tested using an integrated behavioral framework based on the Theory of Planned Behavior (TPB), Tax Morale Theory (TM), and the Slippery Slope Framework (SSF). This model examines the effects of taxpayers' perceptions of GST 2.0 slab rationalization (5% and 18%) on tax morale and voluntary tax compliance behavior, as well as the moderating role of trust in tax authorities. The research has a quantitative, cross-sectional design, with the primary survey data being used on MSMEs in automobile industrial clusters in Tamil Nadu.

### 3.1 Sample and sampling technique

Primary data were gathered among the key stakeholders, i.e., Owners or Proprietors, Partners or Directors, Managers or Accountants, and Consultants or other senior representatives, who operated throughout the automobile supply chain including manufacturers and dealers, and component suppliers, ancillary units, and logistics providers, in major industrial clusters in Tamil Nadu, such as: Chennai–Oragadam–Sriperumbudur, Hosur, Coimbatore, as the main automotive ecosystem of the state (Government of Tamil Nadu, 2023; Society of Indian Automobile Manufacturers, 2024).

The selection of the respondents was based on the premise that the respondents are the key decision-makers with regard to pricing, GST compliance, and strategic reaction to the changes in tax-policies and that they have to face the economic consequences of compliance decisions. Unlike the accounting professionals who often act as intermediaries, these respondents have direct behavioral and operational experience with the GST compliance issues (Loo et al., 2010; Alm, 2019).

Respondents were asked to evaluate their perceptions and anticipated behavioral responses to the proposed GST 2.0 slab rationalization measures based on publicly available policy announcements, reform proposals, and related policy discussions.

The automobile MSME sector was selected due to its wide exposure to GST, the multi-stage supply chains and high sensitivity to slab rationalization. Tamil Nadu offers an adequate empirical context, as it has strong background of automobile industry and a great number of MSME.

Following contemporary PLS-SEM guidelines, the sample size should provide adequate statistical power for estimating the structural model (Hair et al., 2019). With 24 measurement indicators and multiple mediation and moderation effects, a minimum sample exceeding 200 observations was considered adequate. The final sample of 400 valid responses substantially exceeded this requirement, thereby ensuring sufficient statistical power and robust model estimation.

The use of purposive sampling was based on the fact that there was no extensive public database of GST-registered automobile MSMEs. This methodological option is also suitable when it comes to behavioral-taxation studies, which require a particular regulatory experience on the part of the respondents (Etikan et al., 2016; Loo et al., 2010). Structured questionnaire was used in collection of data through online and offline methods in major automobile hubs of Tamil Nadu.

### 3.2 Conceptualization and measurement of variables

The proposed investigation will follow a latent-variable modeling framework created on the assumption that the most essential behavioral constructs are not observable but will, instead, have to be inferred by a variety of indicators (Ajzen, 1991; Hair et al., 2019). In the proposed research model, seven latent constructs have been included and these constructs represent the major aspects of the integrated behavioral framework. The endogenous variable is voluntary tax compliance behavior, and thus it summarizes the actual compliance behavior of the MSME taxpayers in the context of GST 2.0 slab rationalization. The exogenous constructs comprise attitude toward GST compliance, subjective norms, and perceived behavioral control, which represent the key cognitive dimensions of the TPB framework influencing tax morale and voluntary tax compliance behavior (Hair et al., 2019).

TM is presented as a mediating factor that explains how taxpayers' cognitive evaluations are translated into voluntary tax compliance behavior through intrinsic moral motivation (Torgler, 2007; Alm, 2019). Furthermore, Trust in tax authorities is included as a moderating construct based on the SSF, strengthening the relationships between TPB constructs and tax morale (Kirchler et al., 2008).

All constructs were operationally defined based on established behavioral taxation theories, thereby ensuring conceptual validity and theoretical coherence within the GST 2.0 context (Ajzen, 1991).

### 3.3 Questionnaire design

The data were collected via a structured self-administered questionnaire, which was developed in accordance to the already existing literature on behavioral tax compliance (Hair et al., 2019). The questionnaire was divided into three parts. The introduction section outlined the purpose of the research and gave a guarantee on anonymity of the respondents, confidentiality and voluntary participation. The second section gathered firm specific and respondent specific data that is relevant to GST, including the firm age and size, the respondent position, GST compliance experience and the city of operation in the Indian automobile MSME industry.

The third section consisted of measurement items in the latent constructs outlined in the conceptual framework. Twelve items were used to measure the TPB constructs of attitude toward GST compliance, subjective norms, and perceived behavioral control (four items each) (Ajzen, 1991; Bobek et al., 2007). The measure of TM was conducted using four modified questions that indicated intrinsic motivation and moral obligation (Torgler, 2007; Alm, 2019). They measured trust in tax authorities on four items that summed up the ideas of fairness, transparency, and institutional integrity (Kirchler et al., 2008; Gangl et al., 2015). The voluntary tax compliance behavior was measured using four self-reported questions (Bobek and Hatfield, 2003).

On the whole, the instrument consisted of 24 items and each of them was rated using a five-point Likert scale (Likert, 1932). All measurement items were adapted from validated scales reported in prior tax-compliance literature and subsequently contextualized to the GST 2.0 environment. The clarity and content validity of the items were confirmed through a pilot study involving 30 respondents. Table 1 presents the operability indicators, research variables, theoretical foundations, and supporting references used in this study.

**Table 1:** Operability indicator.

Attribute theory	Research variable	Operability indicators	References
TPB theory	Attitude toward GST compliance	Perceived fairness of GST slabs, simplicity of tax rates, economic usefulness, reduction in compliance burden, positive evaluation of GST reforms	Ajzen (1991); Loo et al. (2010); Saad (2014)
TPB theory	Subjective norms	Influence of peers, industry associations, tax consultants, professional expectations	Bobek et al. (2007); Torgler (2007)
TPB theory	Perceived behavioral control	Ease of GST filing, availability of tax knowledge, technological capability, administrative support	Ajzen (1991); Saad (2014)
Tax morale theory	Tax morale	Moral obligation to pay tax, civic responsibility, ethical commitment to compliance	Torgler (2007); Alm et al. (2015)
Slippery slope framework	Trust in tax authorities	Perceived fairness of authorities, transparency, consistency in enforcement, confidence in GST administration	Kirchler et al. (2008); Gangl et al. (2015)
Slippery slope framework	Voluntary tax compliance behavior	Timely filing, accurate reporting, voluntary disclosure, avoidance of aggressive tax practices	Alm (2019); Kirchler et al. (2008)

### 3.4 Data analysis technique

The authors used the Partial Least Squares Structural Equation Modeling (PLS-SEM) with Smart PLS software. PLS-SEM fits especially into research based on behavioral tax (complex models in the study, higher-level constructs, mediation, and moderation) and does not presuppose multivariate normality (Hair et al., 2019, 2021).

The analysis was done in two steps. To test measurement model, first, the indicator loadings, composite reliability (CR), and average variance extracted (AVE) were used. Factor loading threshold values of 0.70 and above, CR of 0.70 to 0.95 and AVE of 0.50 and above were used (Fornell and Larcker, 1981; Hair et al., 2019). Second, the path coefficients, coefficient of determination (*R* 2), effect size (*f* 2) and predictive relevance (*Q* 2) were used to evaluate the model. To determine whether the effects are direct, mediating and moderating, bootstrapping with 5,000 resamples was performed.

### 3.5 Assessment of common method bias

As the data were collected through a single self-reported questionnaire, Harman's single-factor test was employed to assess the potential influence of common method bias. The first unrotated factor accounted for 32.754% of the total variance, which is below the recommended threshold of 50% (Podsakoff et al., 2003). Accordingly, common method bias is unlikely to pose a significant threat to the validity of the study findings.

## 4 Result and analysis

The chapter contains the empirical analysis using the Partial Least Squares Structural Equation Modeling (PLS-SEM) in a two-stage phenomenon. It outlines, first, the profile of Micro, Small, and Medium Enterprise (MSME) respondents who are also working in the automobile industry in India and second, measure's reliability and validity and lastly, it tests the structural model to determine the direct, mediating and moderating impacts of GST 2.0 slab rationalization on the perceptions and behavioral reactions of Indian taxpayers.

### 4.1 Respondents' demographic features

The demographic and firm-specific features of the respondents presented in Table 2 would give the overview of the composition of the samples and denote the contextual base of the empirical analysis. All in all, the profile indicates that the respondents are in the right position to provide informed perceptions and behavioral answer to GST 2.0 slab rationalization in the Indian automobile MSME industry.

**Table 2:** Demographic profile of respondents.

Characteristics	Category	Frequency (*n* = 400)	Percentage (%)
**Gender**	Male	226	56.5
	Female	164	41.0
	Others	10	2.5
**Age (years)**	18–25	64	16.0
	26–35	142	35.5
	36–45	118	29.5
	46–55	56	14.0
	56 or above	20	5.0
**Marital status**	Married	258	64.5
	Unmarried	142	35.5
**Educational qualification**	SSLC	22	5.5
	HSC	74	18.5
	Graduate	198	49.5
	Postgraduate	92	23.0
	Professional/others	14	3.5
**Position in enterprise**	Owner/proprietor	182	45.5
	Partner/director	86	21.5
	Manager/accountant	94	23.5
	Consultant/other	38	9.5
**Nature of enterprise (MSME classification)**	Micro enterprise	168	42.0
	Small enterprise	142	35.5
	Medium enterprise	90	22.5
**Type of business activity**	Manufacturing	146	36.5
	Trading/dealership	158	39.5
	Automobile Ancillary services	96	24.0
**Industrial cluster**	Chennai region (including Oragadam and Sriperumbudur)	180	45
	Hosur	90	22.5
	Coimbatore	80	20
	Other districts	50	12.5
**Years of business operation**	Less than 5 years	88	22.0
	5–10 years	172	43.0
	11–20 years	104	26.0
	Above 20 years	36	9.0
**Annual turnover (₹)**	Below ₹50 lakh	124	31.0
	₹50 lakh–₹5 crore	172	43.0
	₹5 crore–₹50 crore	104	26.0
**GST registration status**	Regular scheme	286	71.5
	Composition scheme	114	28.5
**GST registration duration (years)**	Less than 2 years	82	20.5
	2–5 years	176	44.0
	6–10 years	112	28.0
	Above 10 years	30	7.5
**Frequency of GST return filing**	Monthly	244	61.0
	Quarterly	156	39.0

The sample is fairly balanced, with male respondents (56.5 percent) and female ones (41.0 percent) exactly equal. The age distribution is skewed toward economically active age groups, where 35.5% of the respondents were aged 26–35 years and 29.5% were aged 36–45 years, and thus, this indicates strong entrepreneurial and managerial activity. Most of the respondents are married (64.5%), which implies that they have a stable socio-economic background contributing to the long-term business planning and compliance decisions.

The education level of the sample is high, and almost half of the respondents have graduate (49.5%) Such a high degree of education also means that there is enough understanding of the complicated tax systems and legislations within the GST regime. In terms of businesses, the sample will include those in the various decision-making capacities and this includes the owners or proprietors (45.5%), the partners/ directors (21.5%) and the managerial/accounting staff (23.5%) hence guaranteeing the sample to provide knowledgeable answers to the GST compliance. The greatest portion (42.0%) is comprised of micro enterprises (35.5% 22.5%), then large (small and medium enterprises), respectively. Out of the 400 respondents in the micro, small, and medium enterprise sector, 45% were located in Chennai, 22.5% in Hosur and 20% in Coimbatore and hence this represented very strong representation in the main automotive canters in Tamil Nadu.

The respondents will be spread to manufacturing, trading or dealerships activities, and automobile ancillary services, which will increase representativeness within the value chain. The majority of enterprises are between 5 and 10 years old (43.0–10 years) and it means that they have been exposed to the implementation of GST and subsequent reforms. The fact that regular GST registration (71.5%) and monthly filing of returns (61.0%) are the most common also points to a sample of compliance-intensive individuals. Altogether, these features prove the appropriateness of the sample in studying behavioral reactions to GST slab rationalization.

### 4.2 Evaluation of the measurement model

To test the validity and reliability of the latent constructs, the measurement model was put through validation before structural relationship were tested. The evaluation included indicator's reliability, internal consistency reliability, convergent validity, discriminant validity, collinearity diagnostics and a general model review following acceptable PLS-SEM model standards (Hair et al., 2019; Sarstedt et al., 2022).

#### 4.2.1 Indicator reliability

**Figure 2:**
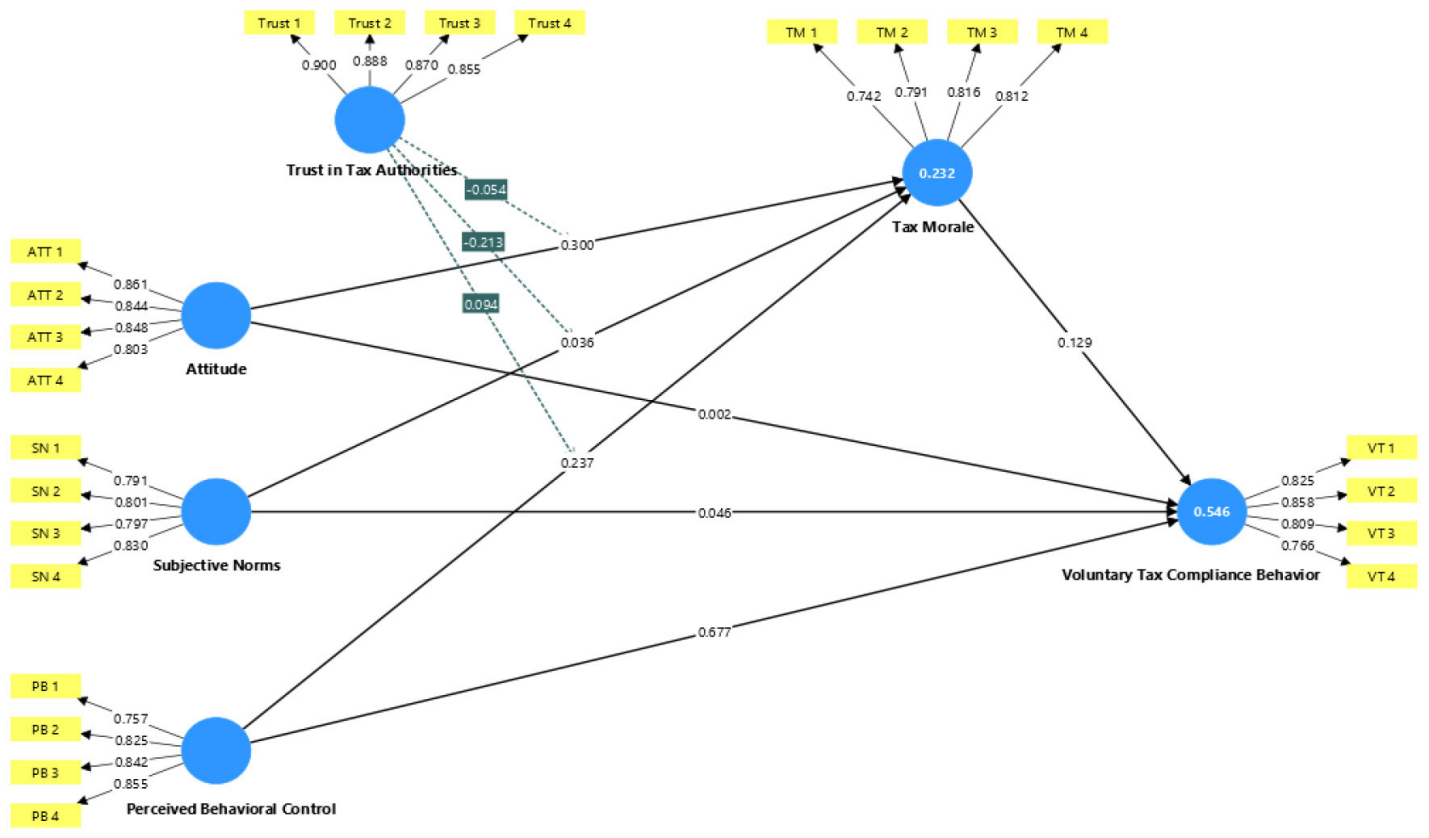
Measurement model diagram.

The standardized outer loadings were evaluated as a measure of the reliability of the indicators. Table 3 and Figure 2 document that the loadings of all indicators are inside the range of 0.742 to 0.900 which is more than the recommended minimum load of 0.70 (Hair et al., 2019). This finding shows that every item explains a significant percentage of the variance in its construct. Therefore, there was no indicator that had to be excluded because all the loadings were within the accepted reliability level.

**Table 3:** Outer loadings of latent constructs.

Construct	Indicator	Outer loading	Status
Attitude	ATT 1	0.861	Passed
	ATT 2	0.844	Passed
	ATT 3	0.848	Passed
	ATT 4	0.803	Passed
Subjective norms	SN 1	0.791	Passed
	SN 2	0.801	Passed
	SN 3	0.797	Passed
	SN 4	0.830	Passed
Perceived behavioral control	PB 1	0.757	Passed
	PB 2	0.825	Passed
	PB 3	0.842	Passed
	PB 4	0.855	Passed
Tax morale	TM 1	0.742	Passed
	TM 2	0.791	Passed
	TM 3	0.816	Passed
	TM 4	0.812	Passed
Trust in tax authorities	TRUST 1	0.900	Passed
	TRUST 2	0.888	Passed
	TRUST 3	0.870	Passed
	TRUST 4	0.855	Passed
Voluntary tax compliance behavior	VT 1	0.825	Passed
	VT 2	0.858	Passed
	VT 3	0.809	Passed
	VT 4	0.766	Passed

#### 4.2.2 Internal consistency reliability and convergent validity

Internal consistency reliability was determined through the use of Cronbach alpha (α) and composite reliability (CR). Cronbach alpha as in Table 4 was found to be 0.801 to 0.902 and CR was found to be 0.870 to 0.931 which is higher than the traditional values of 0.60 and 0.70, respectively (Hair et al., 2019).

**Table 4:** Reliability and convergent validity statistics.

Construct	Cronbach's alpha	Composite reliability (rho_a)	Composite reliability (rho_c)	Average variance extracted (AVE)
Attitude	0.861	0.869	0.905	0.705
Subjective norms	0.822	0.834	0.880	0.648
Perceived behavioral control	0.838	0.842	0.892	0.673
Tax morale	0.801	0.806	0.870	0.625
Trust in tax authorities	0.902	0.912	0.931	0.771
Voluntary tax compliance behavior	0.831	0.832	0.888	0.664

The evaluation of convergent validity was through average variance extracted (AVE). The AVE scores of all constructs were found to be between 0.625 and 0.771, which is higher than the minimum criterion of 0.50 (Fornell and Larcker, 1981). All of these findings endorse sufficient internal consistency reliability and convergent validity of all the latent constructs.

#### 4.2.3 Discriminant validity

The HTMT values presented in Table 5 are all below the recommended threshold of 0.90, providing evidence of adequate discriminant validity (Henseler et al., 2015). Although the relationships between Attitude and Trust (0.880) and Subjective Norms and Trust (0.869) are relatively high, these values remain within acceptable limits and are theoretically justifiable given the conceptual interconnectedness of behavioral and institutional determinants of tax compliance. Furthermore, the square roots of the AVE values exceed the corresponding inter-construct correlations (Table 6), satisfying the Fornell–Larcker criterion (Fornell and Larcker, 1981). Collectively, these findings confirm that the constructs exhibit sufficient empirical distinctiveness while maintaining theoretical coherence within the proposed framework.

**Table 5:** HTMT results.

Construct	ATT	PB	SN	TM	Trust	VT	Trust x ATT	Trust x SN	Trust x PB
**ATT**									
**PB**	0.140								
**SN**	0.848	0.203							
**TM**	0.383	0.385	0.274						
**Trust**	0.880	0.101	0.869	0.257					
**VT**	0.186	0.869	0.234	0.431	0.14				
**Trust x ATT**	0.154	0.183	0.173	0.238	0.143	0.205			
**Trust x SN**	0.152	0.141	0.08	0.300	0.074	0.160	0.614		
**Trust x PB**	0.124	0.090	0.115	0.046	0.065	0.150	0.253	0.221	

**Table 6:** Discriminant validity assessment using the Fornell–Larcker criterion.

Construct	ATT	SN	PB	TAX	TRUST	VT
Attitude (ATT)	**0.840**					
Subjective norms (SN)	0.121	**0.821**				
Perceived behavioral control (PB)	0.716	0.168	**0.805**			
Tax morale (TM)	0.324	0.316	0.234	**0.791**		
Trust in tax authorities (TRUST)	0.771	0.084	0.755	0.225	**0.878**	
Voluntary tax compliance behavior (VT)	0.159	0.726	0.192	0.354	0.120	**0.815**

Bold values on the diagonal represent the square root of the Average Variance Extracted (AVE) for each construct, in accordance with the Fornell–Larcker criterion for assessing discriminant validity.

#### 4.2.4 Collinearity diagnostics

Collinearity had been assessed using the Variance Inflation Factor (VIF). Table 7 provides VIFs between 1.52 and 3.60, all of which are significantly less than the conservative value of 5.0 (Hair et al., 2019). These findings show that multicollinearity is not a relevant issue and it does not skew the estimation of the measurement model.

**Table 7:** Collinearity assessment (VIF).

Indicators	VIF
ATT 1	2.132
ATT 2	2.043
ATT 3	2.022
ATT 4	1.893
SN 1	1.966
SN 2	1.551
SN 3	1.55
SN 4	2.212
PB 1	1.615
PB 2	1.867
PB 3	2.084
PB 4	2.131
TM 1	1.57
TM 2	1.742
TM 3	1.765
TM 4	1.812
TRUST 1	3.595
TRUST 2	2.906
TRUST 3	2.584
TRUST 4	2.023
VT 1	2.061
VT 2	2.325
VT 3	1.794
VT 4	1.52

#### 4.2.5 Model fit indices

Standardized Root Mean Square Residual (SRMR) was used as the measure of goodness-of-fit of the model. Table 8 indicates that SRMR values of the saturated model stand at 0.060, and the estimated model stand at 0.061, which are less than the traditional value of 0.10, and thus give a good fit. The complementary fit statistics, d_ULS, d G, 2 and NFI, support the quality of the model as shown in reference Henseler et al., 2016.

**Table 8:** Model fit indices.

Fit index	Saturated model	Estimated model
SRMR	0.060	0.061
d_ULS	1.098	1.113
d_G	0.415	0.418
Chi-square	985.012	995.003
NFI	0.827	0.825

### 4.3 Structural model assessment

A critical review of the measurement model had been conducted to determine the proposed relationships among the latent constructs by the structural model. The importance and strength of path coefficients, explanatory power (*R*^2^), mediation and moderation, and the effect of control variables are all considered in the process of assessing structural models using PLS-SEM (Hair et al., 2019). The results will be summarized in Tables 8–12.

**Table 9:** Structural path coefficients and hypothesis testing.

Hypothesis	Structural Path	β (path coefficient)	*t*-value	*p*-value	Hypothesis support
H1a	Attitude → Voluntary tax compliance behavior	0.002	0.037	0.971	Not supported
H1b	Subjective Norms → voluntary tax compliance behavior	0.046	0.828	0.408	Not supported
H1c	Perceived behavioral control → Voluntary tax compliance behavior	0.677	16.291	< 0.001	Supported
H2a	Attitude → Tax morale	0.300	3.931	< 0.001	Supported
H2b	Subjective Norms → Tax morale	0.036	0.438	0.662	Not supported
H2c	Perceived behavioral control → Tax morale	0.237	4.089	< 0.001	Supported
H3	Tax morale → Voluntary tax compliance behavior	0.129	2.845	0.004	Supported

**Table 10:** Coefficient of determination (*R*^2^).

Endogenous construct	*R* ^2^	Interpretation
Tax morale	0.232	Moderate explanatory power
Voluntary tax compliance behavior	0.546	Substantial explanatory power

**Table 11:** Mediation analysis results.

Indirect path	Indirect effect (β)	*t*-value	*p*-value	Mediation type
Attitude → Tax morale → Voluntary tax compliance behavior	0.039	2.27	0.023	Partial mediation
Subjective norms → Tax morale → Voluntary tax compliance behavior	0.005	0.412	0.681	No mediation
Perceived behavioral control → Tax Morale → Voluntary tax compliance behavior	0.031	2.237	0.025	Partial mediation

**Table 12:** Moderation analysis results.

Moderating Effect	Interaction β	*t*-value	*p*-value	Result
Trust in tax authorities × Attitude → Tax morale	−0.054	0.647	0.518	Not supported
Trust in tax authorities × Subjective Norms → Tax morale	−0.213	3.082	0.002	Supported
Trust in tax authorities × Perceived behavioral control → Tax morale	0.094	1.63	0.103	Not supported

#### 4.3.1 Direct effects and hypothesis testing

The path coefficients (c) between the two variables were tested to establish direct relationships in the conceptual framework at standardized path coefficients (c), *t*-values, and *p*-values which were bootstrapped as shown in Table 9.

The findings suggest that the direct impact of Attitude on Voluntary Tax Compliance Behaviour (β = 0.002, *p* = 0.971) is not significant enough to suggest that positive attitudes can be converted into the corresponding behaviour on their own. On the same note, there is no significant effect of Subjective Norms on voluntary tax compliance behaviour (β = 0.046, *p* = 0.408), indicating that social pressure has only a minor direct influence on compliance decision-making.

On the contrary, Perceived Behavioral Control is positively associated with voluntary tax compliance behaviour (β = 0.677, *p* < 0.001), indicating that individuals' perceived ability and control are significantly associated with voluntary tax compliance behaviour. In terms of Tax Morale, Attitude (β = 0.300, *p* < 0.001) and Perceived Behavioral Control (β = 0.237, *p* < 0.001) are significant predictors of tax morale, whereas Subjective Norms do not play a significant role (β = 0.036, *p* = 0.662). Moreover, Tax Morale has a positive impact on voluntary tax compliance behaviour (β = 0.129, *p* = 0.004), confirming that it is a major psychological driver connecting individual perceptions with final compliance behaviour.

Altogether, the results support the hypotheses, to some extent, and highlight the crucial role of the perceived behavioral control and tax morale in describing voluntary tax compliance behavior.

#### 4.3.2 Coefficient of determination (R^2^)

Structural model explanatory power was measured with the help of the coefficient of determination (*R*^2^) of the endogenous constructs. As presented in Table 10, the model explains 23.2% of the variance in Tax Morale, which is an indication that it explains moderate amount of variance. Besides this, the model explains 54.6% of the variance in Voluntary Tax Compliance Behavior (*R*^2^ = 0.546) and the model will have significant explanation capabilities.

The findings indicate that the proposed predictors have a significant value in explaining voluntary tax compliance behavior with tax morale being moderately explained by the model. In line with other existing studies on behavioral topic, other psychological and situational reasons might also contribute to tax morale and compliance decisions (Hair et al., 2019).

#### 4.3.3 Mediation analysis

In order to test the hypothesis of the mediation of the relationships between the antecedent variables and voluntary tax compliance behavior, the mediation analysis was carried out through bootstrapped indirect effects. Table 11 provides the results.

The relationship between Attitude and voluntary tax compliance behaviour via Tax Morale is statistically positive and significant (β = 0.039, *t* = 2.270, *p* = 0.023). Both the direct and indirect effects are substantial; therefore, the relationship demonstrates partial mediation. Similarly, Perceived Behavioral Control shows a significant indirect effect through Tax Morale (β = 0.031, *t* = 2.237, *p* = 0.025), confirming partial mediation. In contrast, the indirect effect of Subjective Norms through Tax Morale is not significant (β = 0.005, *p* = 0.681), indicating no mediation. These findings suggest that Tax Morale serves as an important psychological mechanism linking individual perceptions to voluntary tax compliance behaviour.

#### 4.3.4 Moderation analysis

The moderation analysis was conducted to examine the moderating role of trust in tax authorities in the relationships between TPB constructs and tax morale. The effects of interaction were measured in terms of product-indicator terms and Table 12 provides the results.

The interaction effect between trust in tax authorities and subjective norms was negative and significant (β = −0.213, *t* = 3.082, *p* = 0.002), indicating that trust in tax authorities moderates the relationship between subjective norms and tax morale. The negative coefficient suggests that higher trust is associated with a weaker influence of social pressure on tax morale.

Conversely, the interaction effects involving attitude (β = −0.054, *p* = 0.518) and perceived behavioral control (β = 0.094, *p* = 0.103) were not statistically significant; therefore, H5a and H5c were not supported. Overall, the results indicate that trust in tax authorities moderates only the relationship between subjective norms and tax morale, while no significant moderating effects were observed for attitude or perceived behavioral control.

## 5 Discussion

This part analyses the PLS-SEM findings against the hypotheses put forward in the paper based on the Theory of Planned Behavior (TPB Theory), Tax Morale Theory (TM Thoery), and Slippery Slope Framework (SSF). A combination of these views describes the role of attitudes, social impacts, perceived control, moral motivation and institutional trust in facilitating voluntary tax compliance behavior in the GST 2.0 reforms.

### 5.1 TPB constructs and voluntary compliance behavior (H1a, H1b, H1c)

The current research based on the TPB Theory was aimed at exploring the direct relationship between attitude, the subjective norms, and the perceived behavioral control to voluntary tax compliance behavior among MSMEs. The empirical evidence indicates that attitude (H1a) and subjective norms (H1b) are not significantly associated with voluntary tax compliance behavior, whereas perceived behavioral control (H1c) is positively associated with voluntary tax compliance behavior. As a result, the H1a and H1b hypotheses are rejected, but the H1c hypothesis is supported. These findings suggest that positive evaluations of GST compliance and perceived social pressure alone cannot be used to trigger compliant behavior. This result is consistent with earlier studies that indicated that attitudinal and normative effects could be mitigated in the complex tax situations that are marked by procedural ambiguities and administrative burdens (Kirchler et al., 2008; Loo et al., 2010). On the other hand, the high influence of perceived behavioral control shows the significance of the perceived ability of the taxpayers to comply which includes their understanding of GST regulations, filing processes and technological requirements. When MSMEs perceive greater control over compliance processes, they tend to report higher levels of voluntary compliance regardless of their own attitude or social pressures. This finding is consistent with prior GST compliance studies that identified compliance costs, procedural complexity, GST knowledge, and technological readiness as important determinants of compliance behavior among Indian businesses (Kapoor and Singh, 2025; Sharma et al., 2023; Garg et al., 2024a,b,c,d). The present study extends this literature by demonstrating that perceived behavioral control represents an important psychological mechanism through which these compliance capabilities influence voluntary tax compliance behavior.

Accordingly, compliance decisions under GST 2.0 appear to be primarily capability-driven rather than norm-driven. Given the administrative complexity of GST compliance, MSMEs may place greater emphasis on their ability to comply than on social expectations, highlighting perceived behavioral control as a key behavioral mechanism. In terms of the TPB Theory, the result supports the claim that behavioral enactment in taxation contexts is mainly motivated by perceived practicability instead of moral approval or sense of normativity (Ajzen, 1991; Alm, 2019).

### 5.2 TPB constructs and tax morale (H2a, H2b, H2c)

The empirical findings support H2a and H2c, indicating that attitude toward GST compliance and perceived behavioral control are positively associated with tax morale among automobile MSMEs. Contrarily, the data do not support hypothesis H2b, which states that subjective norms positively affect the TM. These results are congruent with TM Theory, which suggests that internalized values, personal considerations, and fairness perceptions are closely associated with taxpayers' moral motivation.

In a TPB Theory view, positive attitudes to GST reforms would increase intrinsic willingness to comply by strengthening the beliefs about legitimacy and fairness, whereas the higher perceived behavioral control would boost the level of confidence in dealing with compliance demands (Ajzen, 1991; Loo et al., 2010). The lack of significance of subjective norms indicates that in a complex regulatory context like GST, subjective motivations of compliance are less determined by social expectations, and more sensitive to personal cognitive evaluations and administrative resources. The SSF that assumes the voluntary compliance to be largely motivated by trust-based motivation over normative pressure in the presence of institutional complexity also supports this interpretation (Kirchler et al., 2008; Gangl et al., 2015). On the whole, the results suggest that the level of TM in MSMEs is more closely associated with attitudinal and control-related elements, as opposed to social norms. While previous GST studies primarily focused on operational and compliance-related factors, the present findings suggest that taxpayers' moral motivation constitutes an additional behavioral mechanism linking GST-related perceptions to compliance behavior (Kapoor and Singh, 2025; Sharma et al., 2023; Garg et al., 2024a,b,c,d).

### 5.3 Tax morale to voluntary compliance behavior (H3)

In line with hypothesis H3, the findings indicate that TM is positively associated with voluntary tax compliance behavior, suggesting that taxpayers with stronger intrinsic moral motivation, civic responsibility, and ethical commitment tend to report higher levels of voluntary GST compliance. This is an important finding that supports the key hypothesis of TM theory that does not consider compliance decision making to rely on the enforcement systems but its decision-making processes are extensively embedded in the internalized norms and moral judgments (Torgler, 2007; Frey and Torgler, 2007).

By following the TPB Theory, TM Theory acts as an intermediary motivational factor that mediates positive attitudes and belief in control into real compliance behavior (Ajzen, 1991). In addition, the SSF indicates that an increased moral commitment increases the trust-based compliance and thus minimizes the use of coercive compliance (Kirchler et al., 2008). The results hence affirm that voluntary compliance of MSMEs in terms of tax compliance is significantly enhanced when taxpayers believe that they are compelled to comply with taxation policies imposed by the government instead of viewing obligation as morally obligatory.

### 5.4 Mediating role of tax morale (H4)

The mediation analysis shows that the relationship between attitude and perceived behavioral control as well as voluntary tax compliance behavior is partially mediated by TM, thus supporting H4a and H4c, but not H4b. Such findings have theoretical implications since they explain the psychological process through which cognitive evaluations are translated into voluntary tax compliance behavior. Although the positive attitude toward the GST compliance and a high level of perceived behavioral control have direct impacts on the compliance behavior, the indirect impact through TM highlights the importance of intrinsic moral motivation.

It means that partial mediation implies that attitudinal and control-based variables contribute to compliance behavior by direct effects of indirect effects in which they strengthen the inner moral duty and civic responsibility of taxpayers. The positive attitudes and perceived control increase voluntary compliance when there is amplification of the attitudes; on the other hand, lower levels of moral motivation may weaken the association between taxpayers' cognitive evaluations and voluntary compliance behavior. This is in line with the TM theory which argues that internalized norms and ethical principles are essential in ensuring voluntary compliance in addition to instrumental reasoning (Torgler, 2007; Frey and Torgler, 2007).

In the TPB Theory, TM may function as a motivational mechanism linking taxpayers‘ cognitive evaluations with voluntary compliance behavior (Ajzen, 1991). Besides, the SSF suggests that high TM enhances compliance on trust, thus reducing the reliance on the deterrence mechanisms (Kirchler et al., 2008; Gangl et al., 2015). Overall, the results suggest that TM is a critical psychological channel, which links TPB constructs and voluntary tax compliance behavior. This finding extends prior GST compliance research by demonstrating that the influence of compliance-related capabilities and perceptions operates not only directly but also indirectly through taxpayers' moral commitment and sense of civic responsibility.

### 5.5 Moderating role of trust in tax authorities (H5)

The moderation analysis revealed that trust in tax authorities significantly moderated the relationship between subjective norms and tax morale, thereby supporting H5b. The negative interaction effect suggests that higher levels of trust are associated with a weaker influence of subjective norms on tax morale. This finding indicates that taxpayers who possess greater confidence in tax authorities may rely less on peer expectations and normative pressures when forming moral motivations toward tax compliance. In contrast, the moderating effects involving attitude and perceived behavioral control were not statistically significant; therefore, H5a and H5c were not supported.

This finding provides behaviorally relevant insights into the role of institutional trust in shaping the relationship between subjective norms and tax morale. Taxpayers who perceive tax authorities as fair, transparent, and effective may rely more on institutional legitimacy and less on peer expectations or social pressures when forming moral motivations toward tax compliance (Tyler, 2006; Kirchler et al., 2008). Conversely, in low-trust environments, social influences may play a more prominent role in shaping tax morale, as taxpayers may depend more heavily on normative cues when evaluating their compliance obligations. This finding is consistent with recent behavioral research suggesting that trust serves as an important mechanism through which individuals interpret information and form decision outcomes in uncertain environments (Vijayananth and Saravanabhavan, 2026).

These findings extend the TPB framework by demonstrating that institutional trust is associated with the relationship between subjective norms and tax morale. Consistent with the Slippery Slope Framework, the results suggest higher levels of trust are associated with lower reliance on social pressures by promoting compliance through perceived legitimacy rather than enforcement (Gangl et al., 2015; Feld and Frey, 2007).

### 5.6 A comprehensive analysis and its theoretical consequences

Together, the results provide a unified, mechanism-oriented explanation of voluntary tax-compliance behavior in the case of micro-, small-, and medium-enterprise (MSME) taxpayers. Voluntary tax compliance behavior is associated with attitudes, subjective norms, perceived behavioral control, tax morale, and institutional trust. These findings suggest that compliance is not merely the product of rational calculation but also reflects cognitive, moral, and institutional processes, with tax morale serving as a motivational mechanism and institutional trust shaping the conditions under which social influences affect moral motivation.

The findings extend TPB by demonstrating that cognitive evaluations influence voluntary tax compliance not only directly but also through tax morale, while institutional trust shapes the effectiveness of this process (Ajzen, 1991). Evidence shows that the effects of attitudes are stronger when they support moral judgment, and trust in tax authorities appears to play an important role in case taxpayers rely on internal or external validations (Kirchler et al., 2008; Gangl et al., 2015). As a theory, this integrative framework expounds on the literature in the domain of behavioral tax-compliance in the sense that it demonstrates how moral motivation and trust-based governance are associated with voluntary compliance, especially when it comes to the setting of an emerging economy. Given the cross-sectional nature of the study, the findings should be interpreted as associations rather than definitive causal relationships.

### 5.7 Implications for management and practice

As the empirical data reveals, tax administrators and policymakers cannot afford to appeal to normative or value-based appeals to supplement voluntary tax compliance by micro-enterprises, small and medium-enterprises (MSMEs). Within the framework of GST 2.0, the communication strategies should clearly relate slab rationalization to tangible benefits of compliance such as lower compliance costs, less ambiguity, and increased cash-flow predictability (Evans, 2003; Saad, 2014). An intense moderating effect of trust in tax authorities emphasizes the necessity to interpret and enforce the rules transparently and simplify digital interfaces to increase the credibility of the institution (Gangl et al., 2015). The informational frictions and TM can be mitigated by concrete interventions in the form of the standardization of GST guidance, the support systems of filing returns, and the creation of grievance-redressal mechanisms, thus encouraging the sustainable voluntary compliance in the Indian automotive MSME industry.

### 5.8 Implications for policy and society

Policy wise, the findings highlight the urgent need to maintain stable GST slab buildings, come up with clear interpretative rules, and put up strong anti-evasion measures so as to strengthen MSME trust in the tax system. The standardized communications and open enforcement systems can dilute ambiguity, as well as strengthen TM, thus creating voluntary compliance (Kirchler et al., 2008; Alm, 2019). In addition to this, taxpayer education programmes should combine GST literacy, tax education, and pragmatic compliance support, as recent evidence indicates that tax literacy and service quality significantly enhance voluntary compliance among MSMEs (Munjeyi and Schutte, 2025). At a macro-level, fostering compliance of MSMEs through trust will promote fair revenue raising and make a significant contribution to the sustainability of fiscal governance in the emerging economies.

## 6 Conclusion and future research directions

The research paper examined the relationship between the perception of taxpayers to voluntary tax care behavior, under the taxation of GST 2.0 slab rationalization, among MSMEs in the Indian automobile sector, using a combination of integrated behavioral theory, incorporating the Theory of Planned Behavior (TPB Theory), Tax Morale Theory (TM Theory), and Slippery Slope Framework (SSF). Through the use of the partial least squares structural equation modeling (PLS-SEM) of survey data assumed to have been gathered on MSME taxpayers, the study provides a mechanism-based explanation of compliance behavior under regulatory uncertainty and complexity in the form of reforms.

The results show that positive attitudes toward compliance do not necessarily result in voluntary tax compliance. Rather, the issue is that behavioral outcomes are predominantly conditioned by the TM, which is a highly important mechanism of critical cognitive and moral evaluation and the intention to actual compliance behavior. Such a mediating role may help explain variations in voluntary compliance even in the presence of positive attitudes and perceived behavioral control in the presence of weak intrinsic motivation and perceived fairness. Further, trust in tax authorities emerged as a significant contextual moderator of the relationship between subjective norms and tax morale. These findings are consistent with the central propositions of the SSF, according to which trust and power play a joint role in enabling voluntary compliance.

Comprehensively, the paper shows that the tax compliance of MSMEs under the GST 2.0 is not solely enforcement-based or value-based. Instead, it is an outcome of negotiated behavioral intents, moral reasoning and institutional trust in an emerging economy context. This study provides valuable insights in the design of trust-enhancing and morale-driven tax reforms by offering contextualized empirical data to the literature on behavioral tax and by giving valuable insights to the impact of compliance costs and regulatory change on a segment of the population (the MSMEs) that is disproportionately affected by behavioral taxes.

### 6.1 Key limitations of the study

Despite the empirical and theoretical contributions made in the study, various constraints are present in the study. First, the study employed a cross-sectional design and relied on self-reported data collected from a single source, which may limit the ability to draw causal inferences and may remain susceptible to common method bias despite procedural and statistical remedies. Future studies may employ longitudinal designs to capture changes in taxpayers' behavioral responses to GST reforms over time.

Second, although purposive sampling was appropriate for accessing GST-experienced MSME respondents, the focus on automobile-sector MSMEs in Tamil Nadu may restrict the generalisability of the findings to other sectors or geographical contexts. Future studies may utilize broader industry samples and multiple geographical regions to enhance external validity. Lastly, the study focuses primarily on voluntary compliance behavior and does not explicitly incorporate enforcement-related factors such as audit experiences, penalty perceptions, or regulatory power, which may also influence taxpayer behavior under the GST system.

### 6.2 Future research recommendations

This study can be furthered in a variety of significant directions in the future research. The longitudinal research design might be able to reflect on the change in attitudes, trust and TM of the taxpayers over time since the research design will capture the changes over time and determine whether the GST slab rationalization has any effect on the continued compliance behavior and not just the expressed intentions. Further investigation into the effects that information framing, openness of GST communication, and perceived fairness of slab structures have on behavioral responses, can be conducted by experimental and mixed-method methods. Comparison of sectors, income or country would assist to evaluate the viability of the proposed model in different institutional and regulatory environments. Future research might also distinguish between microenterprises, small enterprises and medium enterprises or combine objective compliance indicators and the measures of perceptions. Besides, the growing presence of digital tax platforms and e-government systems provide the possibility to understand the impact of the technological-mediated trust and administrative effectiveness on voluntary tax compliance.

### 6.3 Trends and future directions

The current research paper shows that more than simply favorable attitudes of taxpayers have to be sustained to ensure that voluntary tax compliance of micro, small, and medium enterprises (MSMEs) that are already under the updated GST 2.0 slab rationalization framework is realized. Compliance behavior is associated with the interaction between cognitive appraisal, perceived economic consequences, and institutional trust. The synthesized behavioral and governance theoretic perspectives provide the empirical evidence with a sound evidentiary foundation to the development of the trust-based, transparent and inclusive tax policies that can help to increase sustained compliance and create the conditions of sustainable revenue mobilization in the automobile MSME sector of India.

## Data Availability

The raw data supporting the conclusions of this article will be made available by the authors, without undue reservation.

## References

[B1] Ajzen I. (1991). The theory of planned behavior. Org. Behav. Hum. Decis. Process. 50, 179–211. doi: 10.1016/0749-5978(91)90020-T

[B2] Alabede J. O. Zainal Affrin Z. Md Idris K. (2011). Individual taxpayers' attitude and compliance behavior in Nigeria: the moderating role of financial condition and risk preference. J. Account. Tax. 3, 91–104. doi: 10.22164/isea.v5i1.54

[B3] Allingham M. G. Sandmo A. (1972). Income tax evasion: a theoretical analysis. J. Public Econ. 1, 323–338. doi: 10.1016/0047-2727(72)90010-2

[B4] Alm J. (2019). What motivates tax compliance?.J. Econ. Surv. 33, 353–388. doi: 10.1111/joes.12272

[B5] Alm J. Bloomquist K. M. McKee M. (2015). On the external validity of laboratory tax compliance experiments. Econ. Inq. 53, 1170–1186. doi: 10.1111/ecin.12196

[B6] Alm J. Torgler B. (2011). Do ethics matter? tax compliance and morality. J. Bus. Ethic. 101, 635–651. doi: 10.1007/s10551-011-0761-9

[B7] Appiah T. Domeher D. Agana J. A. (2024). Tax knowledge, trust in government, and voluntary tax compliance: insights from an emerging economy. Sage Open 14:21582440241234757. doi: 10.1177/21582440241234757

[B8] Bobek D. D. Hatfield R. C. (2003). An investigation of the theory of planned behavior and the role of moral obligation in tax compliance. Behav. Res. Account. 15, 13–38. doi: 10.2308/bria.2003.15.1.13

[B9] Bobek D. D. Roberts R. W. Sweeney J. T. (2007). The social norms of tax compliance: evidence from Australia, Singapore, and the United States. J. Bus. Ethics 74, 49–64. doi: 10.1007/s10551-006-9219-x

[B10] Cummings R. G. Martinez-Vazquez J. McKee M. Torgler B. (2009). Tax morale affects tax compliance: evidence from surveys and an artefactual field experiment. J. Econ. Behav. Org. 70, 447–457. doi: 10.1016/j.jebo.2008.02.010

[B11] Dell'Anno R. (2009). Tax evasion, tax morale and policy maker's effectiveness. J. Socio-Econ. 38, 988–997. doi: 10.1016/j.socec.2009.06.005

[B12] Dimitras A. Fourlas V. Kirchler E. Peppas G. (2025). Drivers of tax compliance: survey evidence from 1,761 Greek micro-firms. J. Behav. Exp. Econ. 119:102480. doi: 10.1016/j.socec.2025.102480

[B13] Etikan I. Musa S. A. Alkassim R. S. (2016). Comparison of convenience sampling and purposive sampling. Amer. J. Theor. Appl. Stat. 5, 1–4. doi: 10.11648/j.ajtas.20160501.11

[B14] Evans C. (2003). Studying the studies: an overview of recent research into taxation operation costs. eJTR 1:64.

[B15] Feld L. P. Frey B. S. (2007). Tax compliance as the result of a psychological tax contract: The role of incentives and responsive regulation. Law Policy, 29, 102–120. doi: 10.1111/j.1467-9930.2007.00248.x

[B16] Fornell C. Larcker D. F. (1981). Evaluating structural equation models with unobservable variables and measurement error. J. Market. Res. 18, 39–50. doi: 10.1177/002224378101800104

[B17] Frey B. S. Torgler B. (2007). Tax morale and conditional cooperation. J. Comparat. Econ. 35, 136–159. doi: 10.1016/j.jce.2006.10.006

[B18] Gangl K. Hofmann E. Kirchler E. (2015). Tax authorities' interaction with taxpayers: a conception of compliance in social dilemmas by power and trust. New Ideas Psychol. 37, 13–23. doi: 10.1016/j.newideapsych.2014.12.001 25859096 PMC4381354

[B19] Garg S. Mittal S. Garg A. (2024a). Navigating GST revenue efficiency challenges: a solution to dilemma of policy makers for enhancing revenue efficiency. J. Indian Bus. Res. 16, 393–409. doi: 10.1108/JIBR-05-2024-0114

[B20] Garg S. Narwal K. P. Kumar S. (2024b). Application of theory of planned behavior on determinants of GST compliance behavior of GST taxpayers: an empirical study from India. J. Tax Reform. 10, 134–148.doi: 10.15826/jtr.2024.10.1.161

[B21] Garg S. Narwal K. P. Kumar S. (2024c). Building the empirical puzzle on impact of macroeconomic determinants on GST revenue: an empirical investigation via ARDL bound test perspective. J. Public Affairs 24:e2947. doi: 10.1002/pa.2947

[B22] Garg S. Narwal K. P. Kumar S. (2024d). Investigating the compliance behavior of GST taxpayers: an extension to theory of planned behavior. J. Public Affairs 24:e2936. doi: 10.1002/pa.2936

[B23] Government of India (2022). Report of the GST Council on Rate Rationalisation and Correction of Inverted Duty Structure. New Delhi: Ministry of Finance, Department of Revenue. Available online at: https://www.gstcouncil.gov.in

[B24] Government of Tamil Nadu (2023). Vision Tamil Nadu 2023, Volume II: Manufacturing and Industrial Development. Chennai: Government of Tamil Nadu.

[B25] Hair J. F. Hult G. T. M. Ringle C. M. Sarstedt M. Danks N. P. Ray S. (2021). Partial Least Squares Structural Equation Modeling (PLS-SEM) Using R: A Workbook (Berlin: Springer Nature), 197. doi: 10.1007/978-3-030-80519-7

[B26] Hair J. F. Risher J. J. Sarstedt M. Ringle C. M. (2019). When to use and how to report the results of PLS-SEM. Eur. Bus. Rev. 31, 2–24. doi: 10.1108/EBR-11-2018-0203

[B27] Hanno D. M. Violette G. R. (1996). An analysis of moral and social influences on taxpayer behavior. Behav. Res. Account. 8, 57–75. doi: 10.2308/BRIA-7709432

[B28] Henseler J. Hubona G. Ray P. A. (2016). Using PLS path modeling in new technology research: updated guidelines. Ind. Manag. Data Syst. 116, 2–20. doi: 10.1108/IMDS-09-2015-0382

[B29] Henseler J. Ringle C. M. Sarstedt M. (2015). A new criterion for assessing discriminant validity in variance-based structural equation modeling. J. Acad. Market. Sci. 43, 115–135. doi: 10.1007/s11747-014-0403-8

[B30] Kapoor S. Singh A. (2025). Issues perceived by business taxpayers regarding implementation of GST in india: scale development and validation. Vision. doi: 10.1177/09722629251379991

[B31] Kirchler E. Hoelzl E. Wahl I. (2008). Enforced versus voluntary tax compliance: the “slippery slope” framework. J. Econ. Psychol. 29, 210–225. doi: 10.1016/j.joep.2007.05.004

[B32] Likert R. (1932). A Technique for the Measurement of Attitudes. Chicago, IL: Archives of Psychology.

[B33] Loo E. C. Evans C. McKerchar M. A. (2010). Challenges in understanding compliance behaviour of taxpayers in Malaysia. Asian J. Bus. Account. 3:2010. doi: 10.2139/ssrn.2128378

[B34] Luttmer E. F. Singhal M. (2014). Tax morale. J. Econ. Perspect. 28, 149–168. doi: 10.1257/jep.28.4.149

[B35] Mallick H. (2026). An assessment of the efficiency of indirect tax revenue mobilisation by indian states: a comparative analysis during VAT and GST regimes. Aust. Econ. Pap. 1–17. doi: 10.1111/1467-8454.70024

[B36] Mebratu A. A. (2024). Theoretical foundations of voluntary tax compliance: evidence from a developing country. Human. Soc. Sci. Commun. 11, 1–8. doi: 10.1057/s41599-024-02903-y

[B37] Muehlbacher S. Kirchler E. Schwarzenberger H. (2011). Voluntary versus enforced tax compliance: empirical evidence for the “slippery slope” framework. Eur. J. Law Econ. 32, 89–97. doi: 10.1007/s10657-011-9236-9

[B38] Mukherjee S. (2020). Performance ASSESSment of Indian GST: State-Level Analysis of Compliance Gap and Revenue Growth (No. 20/301). New Delhi: National Institute of Public Finance and Policy (NIPFP).

[B39] Munjeyi E. Schutte D. P. (2025). Voluntary tax compliance determinants among small and medium enterprises in democratic societies: the role of tax literacy, tax amnesty, tax reward, and service delivery. Cogent Bus. Manag. 12:2526139. doi: 10.1080/23311975.2025.2526139

[B40] OECD (2019) Tax Morale: What Drives People and BUSINESSes to Pay Tax? Paris: OECD Publishing. Available online at: https://www.oecd.org/tax/tax-global/tax-morale.htm

[B41] OECD (2020) Taxation and SMEs: Key Issues and Policy Considerations. Paris: OECD Publishing. Available online at: https://www.oecd.org/tax/tax-policy/taxation-and-smes.html

[B42] Podsakoff P. M. MacKenzie S. B. Lee J. Y. Podsakoff N. P. (2003). Common method biases in behavioral research: A critical review of the literature and recommended remedies. J. Appl. Psychol. 88, 879–903. doi: 10.1037/0021-9010.88.5.879 14516251

[B43] Reserve Bank of India (2025) Report on Trend and Progress of Banking in India 2024–25. Mumbai: Reserve Bank of India.

[B44] Richardson G. (2006). Determinants of tax evasion: a cross-country investigation. J. Int. Account. Audit. Tax. 15, 150–169. doi: 10.1016/j.intaccaudtax.2006.08.005

[B45] Saad N. (2014). Tax knowledge, tax complexity and tax compliance: taxpayers' view. Procedia Soc. Behav. Sci., 109, 1069–1075. doi: 10.1016/j.sbspro.2013.12.590

[B46] Saad N. Ya'u A. (2019). Fairness perceptions and voluntary tax compliance in Nigeria: the moderating role of trust. Glob. Bus. Manag. Rev. (GBMR), 11, 54–76. doi: 10.32890/gbmr2019.11.2.8661

[B47] Sarstedt M. Hair J. F. Pick M. Liengaard B. D. Radomir L. Ringle C. M. (2022). Progress in partial least squares structural equation modeling use in marketing research in the last decade. Psychol. Market. 39, 1035–1064. doi: 10.1002/mar.21640

[B48] Sharma A. Sharma P. Singh J. (2023). Analysing the framework of tax compliance: A study of attitudinal determinants. Metamorphosis 22, 7–17. doi: 10.1177/09726225231170719

[B49] Sniehotta F. F. Presseau J. Araújo-Soares V. (2014). Time to retire the theory of planned behavior. Health Psychol. Rev. 8, 1–7. doi: 10.1080/17437199.2013.869710 25053004

[B50] Society of Indian Automobile Manufacturers (2024). Annual Report 2023–24. New Delhi: Society of Indian Automobile Manufacturers. Available online at: https://www.siam.in/uploads/Know/AnnualReport/26-2023-2024.pdf

[B51] Thakur T. Devi A. D. (2025). GST Reforms 2025 and the new tax regime in India: towards simplified taxation or added complexity. J. Econ. Manag. Trade 31, 94–113. doi: 10.9734/jemt/2025/v31i121375

[B52] Torgler B. (2007). Tax Compliance and Tax Morale: A Theoretical and Empirical Analysis. Cheltenham: Edward Elgar Publishing. doi: 10.4337/9781847207203

[B53] Tumoro D. T. Pandya H. B. (2025). Social psychological drivers and tax revenue for sustainable economic development with the mediating role of tax compliance, evidence from Hossana city, Ethiopia. Discov. Sustain. 6:1062. doi: 10.1007/s43621-025-01728-2

[B54] Tyler T. R. (2006). Why People Obey the Law. Princeton: Princeton university press. doi: 10.1515/9781400828609

[B55] Vijayananth M. Saravanabhavan N. (2026). Beyond algorithmic trust: human–AI interaction competency strengthens AI-driven financial decision-making and financial resilience. Front. Artif. Intell. 9:1867266. doi: 10.3389/frai.2026.1867266 42305940 PMC13265365

